# Perspectives on human adipose tissue: from cellular mechanisms to clinical complications

**DOI:** 10.1007/s00125-026-06735-0

**Published:** 2026-04-24

**Authors:** Mikael Rydén

**Affiliations:** 1 Department of Medicine Huddinge, Karolinska Institutet, Karolinska University Hospital, Stockholm, Sweden; 2 Steno Diabetes Center Copenhagen, Herlev, Denmark

**Keywords:** Adipocytes, Adipogenesis, Depots, Diabetes, Inflammation, Insulin resistance, Obesity, Review, Single-cell transcriptomics

## Abstract

White adipose tissue (WAT) is a remarkably plastic organ integrating metabolic, endocrine and immune functions. In humans, perturbations in adipocyte turnover, size regulation and cellular heterogeneity underpin the transition from metabolic health to obesity-related disease. This review, based on the 2025 Camillo Golgi Prize Lecture, highlights selected mechanistic and translational insights, primarily from our work and that of close collaborators, that have contributed to reshaping our understanding of human adipose biology. ^14^C-dating and clinical studies demonstrate that adipocytes are continuously renewed, and that impaired lipid turnover predisposes to weight gain, insulin resistance and type 2 diabetes. Obesity induces persistent transcriptional and structural alterations, an ‘adipose memory’, that provides a biological basis for understanding the difficulty of maintaining weight loss. Spatial transcriptomics has revealed at least three major adipocyte states in human WAT that differ in insulin responsiveness and lipid-handling capacity, suggesting that adipocyte composition influences depot function and responsiveness to interventions such as weight loss or pharmacological therapies. In addition, depot-specific enrichment of immunomodulatory adipocytes, particularly in epiploic WAT, highlights local adipocyte–immune crosstalk as a contributor to systemic inflammation. Collectively, these advances redefine human WAT as an active driver of cardiometabolic disease and highlight therapeutic strategies targeting adipocyte plasticity and immune–metabolic interactions.

**Graphical Abstract:**

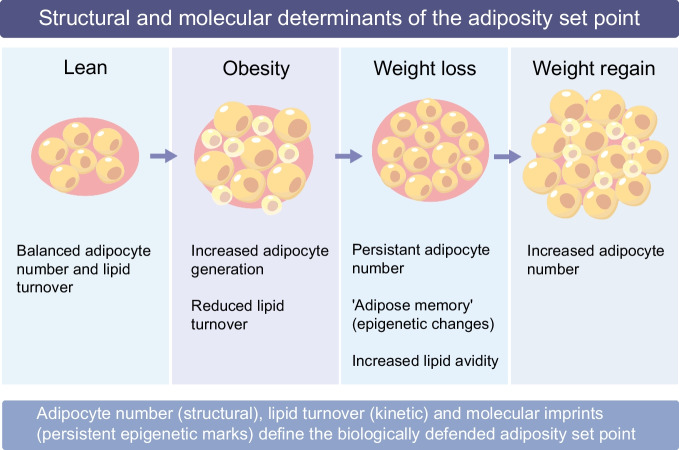

**Supplementary Information:**

The online version contains a slideset of the figures for download available at 10.1007/s00125-026-06735-0.

## The central role of white adipose tissue in metabolic disease

For many years, white adipose tissue (WAT) occupied a rather peripheral position in metabolic science. Long regarded primarily as a passive reservoir for excess energy, it was often considered biologically inert compared with organs such as the liver, muscle or pancreas. Consequently, early mechanistic frameworks for obesity-related disease focused largely on insulin action in classical target tissues, while WAT was viewed mainly as a consequence, rather than a driver, of metabolic dysfunction. This perception has changed fundamentally over the past decades. The recognition that WAT is hormonally active, immunologically complex and highly dynamic has repositioned it at the centre of metabolic research [1]. WAT continuously adapts to nutritional and hormonal cues through coordinated regulation of lipid storage, cellular turnover and intercellular communication [2]. These adaptive processes allow WAT to buffer fluctuations in energy balance and protect other organs from lipid overload. At the cellular level, this adaptability is largely mediated by changes in adipocyte size. During positive energy balance, excess lipids are stored as triacylglycerols within adipocytes, leading to cell enlargement (hypertrophy), whereas weight loss is accompanied by lipid mobilisation and a reduction in adipocyte size. While these size changes are reversible, they are constrained by longer-lasting features of WAT architecture, including adipocyte number and cellular composition. Importantly, when adipocyte lipid storage capacity is exceeded or dysregulated, or when lipid turnover becomes impaired, WAT shifts from a metabolic buffer to a metabolic liability. Impaired regulation of adipocyte size, reduced lipid flux and altered WAT architecture are accompanied by tissue hypoxia, fibrosis and immune cell recruitment, collectively transforming WAT into a site of chronic low-grade inflammation. Such changes are associated with systemic insulin resistance and the development of cardiometabolic disease, highlighting that the quality of WAT function is at least as important as the quantity of stored fat. At the population level, the global rise in obesity has made these insights clinically relevant. Obesity is associated with a wide range of complications, including type 2 diabetes, cardiovascular disease, liver steatosis and several malignancies, yet individuals with similar degrees of adiposity often display markedly different metabolic risk profiles [3, 4]. The concept of metabolically healthy obesity (MHO) illustrates that excess adiposity per se does not inevitably translate into insulin resistance or cardiovascular risk [5]. Although the long-term stability of the MHO phenotype remains debated [6, 7], its existence underscores that qualitative features of WAT modify disease risk beyond fat mass alone. Moreover, fat distribution critically shapes metabolic outcomes. Visceral depots, particularly those draining into the portal circulation, are more strongly associated with dyslipidaemia, hepatic insulin resistance and cardiometabolic disease than subcutaneous depots [8]. Differences in lipolytic activity, adipokine secretion and immune cell composition between depots likely contribute to this divergence. Thus, both quantitative and anatomical characteristics of WAT must be considered when interpreting metabolic risk. Understanding how WAT remodels over time, how distinct adipocyte states contribute to tissue function and why some changes persist even after weight loss has become central to identifying durable strategies to prevent and treat metabolic disease.

## Lipid turnover and the adiposity set point

A central question in obesity research is why excess adiposity, once established, is so resistant to reversal. Early models emphasised energy intake and expenditure, implicitly assuming that WAT passively expands and contracts in response to energy balance [9]. However, human studies over the past two decades have demonstrated that the dynamics of lipid storage and mobilisation within adipocytes, collectively referred to as lipid turnover, are tightly regulated processes that differ markedly between individuals and across metabolic states [2]. While energy intake and expenditure determine short-term energy balance, the capacity of WAT to store and mobilise lipids influences how efficiently small and often imperceptible energy surpluses are retained over time [2]. Impaired adipocyte lipid turnover favours lipid retention rather than mobilisation, thereby biasing body weight trajectories towards gradual weight gain even in the absence of overt hyperphagia. In this framework, WAT dysfunction does not contradict classical energy-balance models but adds a biological layer explaining how small energy imbalances are retained, and potentially reinforced over time.

The use of ^14^C-dating provided the first direct evidence that human adipocytes are continuously renewed throughout adult life, with an mean lifespan of approximately 10 years [10]. These studies established that adipose tissue mass is maintained through a balance between adipocyte formation and death, rather than by static persistence of fat cells. Importantly, using different approaches down to the single-cell level, we could show that individuals living with obesity display an increased rate of adipocyte generation [10, 11]. This results in a higher total number of fat cells that remains largely unchanged following weight loss [12]. In the ‘post-obese’ state, WAT is characterised by many small fat cells, reflecting persistent hyperplasia. We and others have demonstrated that the transcriptional signature [12, 13] of WAT following sustained weight loss differs, and that secretion of the satiety hormone leptin is reduced compared with BMI-matched individuals that have remained weight-stable [14]. Thus, adipocyte number can only increase and, once expanded, defines a long-lasting structural constraint on WAT mass, which may also explain why repeated weight cycling is so detrimental to cardiometabolic health [15–17].

Beyond cell number, the flux of lipids through individual adipocytes emerged as a critical determinant of metabolic health [18–20]. By combining triacylglycerol ^14^C-dating with ex vivo lipolysis assays and long-term clinical follow-up, it became evident that lipid turnover varies widely between individuals and is not simply a consequence of obesity. In cross-sectional analyses, we demonstrated that reduced triacylglycerol turnover and attenuated hormone-stimulated lipolysis are strongly associated with features of the metabolic syndrome (ATP III), dyslipidaemia and insulin resistance, independent of fat mass [21, 22]. Prospective studies revealed that disturbances in lipid turnover precede and predict metabolic disease. Individuals characterised by low stimulated lipolysis and prolonged triacylglycerol residence time were more likely to gain weight over time and to develop impaired glucose tolerance or type 2 diabetes [23]. These observations provided direct evidence that impaired adipocyte lipid mobilisation is not merely a marker of, but a likely contributor to, long-term weight gain and metabolic deterioration. It is important to note that most human lipid turnover studies have been performed in subcutaneous WAT. Visceral depots display higher basal lipolytic activity and direct drainage into the portal circulation, potentially amplifying hepatic exposure to NEFAs, which forms the basis of the classical portal hypothesis [20, 24]. Thus, depot-specific differences in lipid flux may differentially influence systemic insulin resistance and dyslipidaemia.

**Fig. 1:**
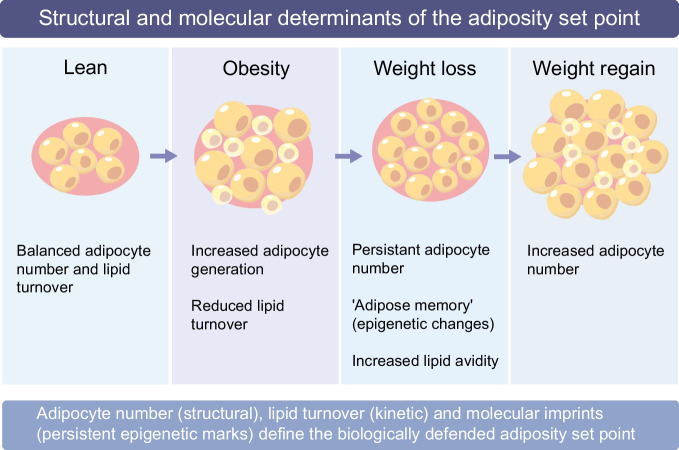
Structural and molecular determinants of the adiposity set point. WAT mass is regulated by interacting structural, kinetic and molecular mechanisms. In the lean state, adipocyte number and lipid turnover are balanced, allowing metabolic flexibility. During obesity development, adipocyte hypertrophy and increased adipocyte generation (newly formed cells depicted in pastel yellow) establish a higher adipocyte number and reduced lipid turnover, shifting the defended adiposity set point upward. Following weight loss, adipocyte number remains elevated despite cell shrinkage, and persistent transcriptional and epigenetic alterations (‘adipose memory’) may promote increased lipid avidity. Upon weight regain, both adipocyte refilling and further adipocyte recruitment contribute to restoration of fat mass. Together, adipocyte number (structural), lipid flux (kinetic) and sustained molecular imprints define the biologically defended level of adiposity and may help to explain the difficulty of maintaining long-term weight loss. This figure is available as part of a downloadable slideset

Together, our findings support the notion of a biologically defined ‘set point’ of adiposity, shaped by both adipocyte number and lipid turnover capacity. Once established, this set point favours lipid retention, rendering weight loss difficult to sustain. Following caloric restriction, adipocyte number remains unchanged, while fat cells become smaller and exhibit hormonal and transcriptional signatures consistent with increased lipid avidity. This state creates a strong biological drive towards weight regain, helping to explain the limited long-term success of lifestyle-based interventions. The observation that even modern incretin therapies do not protect against weight regain after cessation aligns with this notion [25, 26]. Adipocytes do not express GLP-1 receptors, implying that incretin-induced weight-loss effects are not mediated via direct effects on fat cells and, therefore, have no impact on the adipose ‘set point’. From a clinical perspective, these findings emphasise that long-term metabolic risk and weight regain are shaped not only by behaviour but by intrinsic properties of WAT, underscoring the need for interventions that modify adipocyte biology rather than transiently suppress appetite or caloric intake. Altogether, human WAT is a dynamic system with a ‘memory’, in which past nutritional states leave enduring imprints on cellular composition and metabolic function [27]. The concepts of an adiposity ‘set point’ and an ‘adipose memory’ are closely related but not identical. The set point refers primarily to structural and kinetic properties of WAT, such as adipocyte number and lipid turnover capacity, that bias long-term energy storage. In contrast, adipose memory encompasses persistent transcriptional and epigenetic alterations induced by prior nutritional states, which may constrain how adipocytes respond to subsequent interventions. These mechanisms likely interact, with structural features reinforcing molecular imprints and vice versa (Fig. 1). Nevertheless, recognising lipid turnover as a central regulator of adiposity reframes obesity from a simple imbalance of calories towards a disorder of WAT dynamics, in which impaired lipid flux and persistent cellular states limit durable weight loss. This may have important implications for the development of therapies aimed at achieving long-term metabolic improvement rather than transient weight loss.

## From lean to obese: the inflammation hypothesis

The association between obesity and chronic low-grade inflammation in WAT was recognised early on and rapidly became a dominant explanatory model for understanding insulin resistance [28]. Initial studies identified increased expression of proinflammatory cytokines, most notably tumour necrosis factor-α (TNF-α), in WAT from obese mice [29] and insulin-resistant individuals with obesity [30], and subsequent work highlighted macrophage accumulation as a hallmark of metabolically unhealthy fat [31, 32]. These observations gave rise to an immune-centric model in which infiltrating leukocytes were viewed as the primary drivers of adipose tissue dysfunction. While this framework captured important aspects of obese WAT biology, it soon became evident that it was incomplete. Anti-inflammatory strategies targeting immune cells or cytokines yielded only modest metabolic benefits in clinical settings [33–35], suggesting that immune activation alone could not readily explain the link between WAT and cardiometabolic disease. These limitations prompted re-evaluation of the sequence linking excess adiposity to inflammation.

Accumulating mechanistic data instead point to dysfunctional adipocytes as the initiating signal. Adipocyte hypertrophy is a well-established trigger of proinflammatory signalling [36]. Enlarged adipocytes experience mechanical stress and relative hypoxia, leading to chemokine production and immune cell recruitment. Importantly, immune remodelling in WAT involves not only recruited macrophages but also neutrophils [37] and resident immune populations, including innate lymphoid cells, T cells, B cells and mast cells [38], which collectively shape the inflammatory milieu. In this context, inflammation emerges as a downstream consequence of adipocyte metabolic stress rather than its primary cause. As adipocytes enlarge and lose metabolic flexibility, their capacity to safely store and mobilise lipids also becomes compromised. This state is characterised by altered intracellular metabolism, impaired hormone responsiveness and cellular stress, creating a microenvironment that promotes immune activation. Several mechanistic pathways support this adipocyte-first concept. The chemokine monocyte chemoattractant protein-1 (MCP1/CCL2), long viewed as an immune-derived mediator, is also produced by stressed adipocytes and may govern immune cell recruitment into WAT [39]. We and others have shown that alterations in adipocyte glutamine metabolism modulate inflammatory signalling and macrophage recruitment, highlighting how nutrient flux within adipocytes can shape immune responses [40, 41]. In this context, our work has provided additional mechanisms in which adipocyte *CCL2* expression is upregulated in a creatine-dependent manner, again linking cellular energetics to tissue-level inflammation [42, 43].

This shift in perspective has important therapeutic implications. If inflammation in WAT is primarily driven by adipocyte stress and metabolic inflexibility, then targeting immune cells alone is unlikely to restore tissue homeostasis. Instead, interventions that improve adipocyte lipid turnover, metabolic capacity and cellular resilience may indirectly, but more effectively, attenuate inflammation. Such strategies align with data showing that improvements in adipocyte function, whether through weight loss or pharmacological modulation, are accompanied by reduced inflammatory signatures, even in the absence of direct immunosuppression [13, 44, 45]. Thus, the transition from lean to obese WAT can be understood as a progression from metabolically adaptable to functionally constrained adipocytes, with inflammation emerging as a downstream consequence [37]. Reframing WAT inflammation in this way places adipocytes at the centre of disease pathogenesis and provides a coherent explanation for why sustained metabolic improvements require restoration of adipocyte function rather than suppression of immune activation alone.

## Adipocyte heterogeneity revealed by spatial transcriptomics

**Fig. 2:**
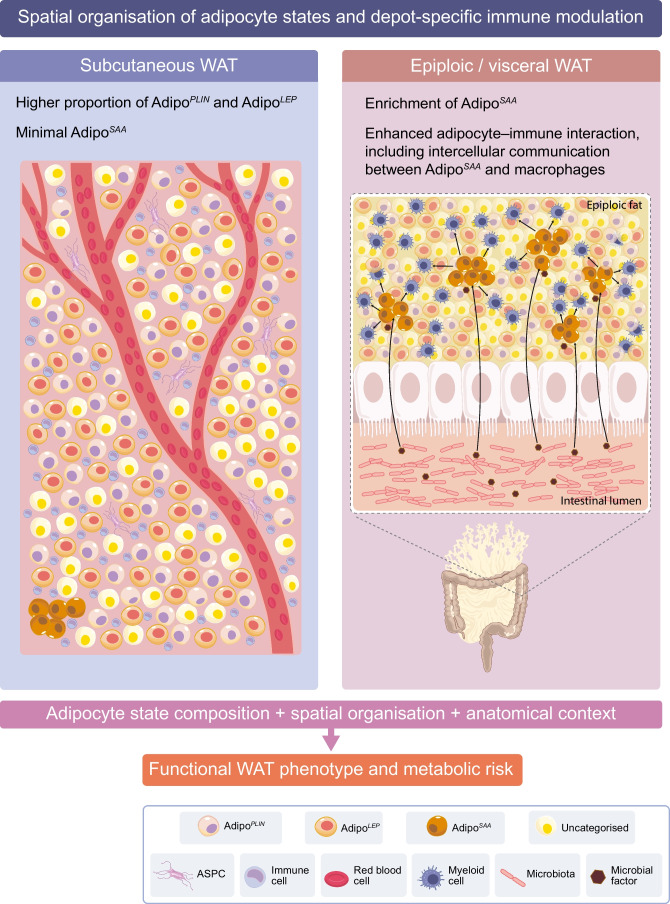
Spatial organisation of adipocyte states and depot-specific immune modulation in human WAT. Human WAT is composed of spatially organised adipocytes in varying states with distinct transcriptional and functional properties. In subcutaneous depots, adipocytes involved in lipid turnover (Adipo^*PLIN*^) and mass sensing (Adipo^*LEP*^) predominate, supporting metabolic buffering and insulin responsiveness. In contrast, epiploic/visceral depots display enrichment of immunomodulatory adipocytes (Adipo^*SAA*^) and enhanced adipocyte–immune cell interactions, potentially influenced by proximity to the intestinal environment. Depot-specific differences in adipocyte state composition and spatial organisation shape local immune tone and contribute to inter-individual variability in metabolic risk. Together, adipocyte heterogeneity and anatomical context determine the functional phenotype of WAT. This figure is available as part of a downloadable slideset

For many years, adipocytes were treated as a largely homogeneous population, with differences in cell size considered the primary source of functional variability. While adipocyte hypertrophy undoubtedly influences metabolic behaviour, this view could not fully explain the marked inter-individual differences in insulin sensitivity, inflammatory tone and response to interventions observed among people with similar adiposity [46]. Advances in high-resolution transcriptomic profiling have now fundamentally revised this assumption. The application of spatial transcriptomics to intact human WAT has revealed that adipocytes segregate into at least three major states (Adipo^*PLIN*^, Adipo^*LEP*^ and Adipo^*SAA*^) with distinct transcriptional and functional profiles [47] (Fig. 2). These adipocytes differ in their capacity for insulin-stimulated glucose uptake, lipid turnover and metabolic responsiveness, demonstrating that functional heterogeneity is an intrinsic property of adipose tissue rather than a secondary consequence of disease. Whether these fat cells originate from distinct progenitor populations or represent plastic differentiation states arising from a common lineage remains an important unresolved question. Several non-mutually exclusive mechanisms may contribute to the emergence and maintenance of these adipocyte states. Prior nutritional exposure or changes in WAT mass may induce stable epigenetic modifications that bias adipocytes towards specific transcriptional programmes. In addition, differences in lipid flux, cellular stress responses or local paracrine signalling from immune and stromal cells could reinforce state-specific gene expression patterns. Importantly, these adipocyte states should not be viewed as rigid or mutually exclusive cell types, but rather as partially overlapping and potentially dynamic phenotypes that may interconvert in response to metabolic or environmental cues.

Notably, these cell-specific differences map onto the lipid turnover framework described above and may translate into inter-individual differences in lipid flux and metabolic risk. Thus, our results suggest that Adipo^*PLIN*^ are enriched in pathways related to lipid turnover, whereas Adipo^*LEP*^ may be involved in sensing changes in WAT mass [47]. Adipo^*SAA*^ display a more inflammatory profile and their potential role is discussed more below in relation to the presence of these states in different WAT depots (Fig. 2). Importantly, these adipocyte states are spatially organised within the tissue. Integration of spatial transcriptomics with single-cell and single-nucleus RNA sequencing has enabled the in vivo definition of adipocyte states that were previously obscured by bulk tissue analyses. As spatial transcriptomics provides complementary information by preserving tissue architecture, it also enables mapping of adipocyte states in relation to vascular structures, extracellular matrix organisation and immune-cell niches. This offers insights into how microenvironmental cues and potential developmental origins contribute to transcriptomic diversity. This multimodal approach has made it possible to link transcriptional identity to functional phenotypes measured directly in humans, including insulin responsiveness during hyperinsulinaemic–euglycaemic clamp studies. These studies indicate that insulin action in WAT is not uniformly distributed across adipocytes, but instead reflects the relative abundance and functional state of specific adipocyte subpopulations.

Adipocyte heterogeneity may also provide a conceptual basis for understanding inter-individual variability in metabolic health. Data from small cohorts indicate that individuals differ substantially in the proportion of insulin-responsive adipocytes present in their subcutaneous WAT, even when matched for BMI or fat mass [47, 48]. Moreover, interventions, such as weight loss or glucose-lowering drugs, may not uniformly shift adipocyte populations towards a more metabolically favourable state. This is supported by observations that some individuals exhibit marked improvements in insulin sensitivity (reflecting adipocyte insulin responsiveness), while others show limited or heterogeneous responses to such interventions, suggesting that pre-existing adipocyte composition may constrain therapeutic outcomes [49]. It is also possible that adipocyte heterogeneity is dynamic and influenced by both nutritional history and pharmacological interventions. Thus, weight loss, incretin-based therapies, and other metabolic or pharmacological treatments may alter adipocyte state distributions, but the direction and magnitude of these changes could vary between individuals. This raises the possibility that adipocyte subtype composition may predict, or even influence, responsiveness to specific interventions, a hypothesis we are currently testing in clinical intervention trials (ClinicalTrials.gov registration nos. NCT01727245 and NCT05501483). Individuals enriched in adipocyte states characterised by high lipid turnover capacity may primarily benefit from interventions aimed at sustaining adipocyte function and preventing maladaptive remodelling, whereas dominance of inflammatory- or stress-associated adipocyte states may identify patients more likely to respond to treatments that indirectly restore adipocyte metabolic flexibility. In this context, transcriptional or circulating markers reflecting WAT cell composition could serve as biomarkers to stratify patients, predict treatment response or guide the choice and timing of metabolic interventions. Although such approaches remain conceptual, they provide a foundation for moving beyond uniform weight-loss strategies towards phenotype-informed metabolic therapy. Collectively, these observations position adipocyte heterogeneity as a key determinant of WAT function. Recognising that WAT is composed of functionally distinct adipocyte populations helps reconcile long-standing discrepancies between adiposity and metabolic risk and provides a mechanistic basis for personalised approaches to metabolic disease treatment.

## Depot-specific immune modulation: insights from studies of visceral depots

WAT is distributed across multiple anatomical depots that differ markedly in developmental origin, vascularisation, innervation and metabolic impact [50, 51]. Classical distinctions between subcutaneous and visceral fat have long been recognised in relation to cardiometabolic risk, yet such binary classifications obscure substantial heterogeneity within visceral depots themselves. Recent transcriptomic and spatially resolved analyses of human WAT now indicate that depot-specific cellular composition and intercellular communication shape local immune tone and systemic metabolic consequences [51]. Comparative profiling of subcutaneous, omental, mesenteric and epiploic WAT has revealed pronounced differences in both adipocyte and stromal cell populations [48]. Among these depots, epiploic WAT, situated in close proximity to the colon, stands out because of its enrichment of Adipo^*SAA*^, characterised by expression of serum amyloid A (*SAA*) genes. These adipocytes are rare in subcutaneous depots but represent a substantial fraction of the adipocyte population in epiploic fat, suggesting strong anatomical and environmental influences on adipocyte identity. Spatial and ligand-receptor analyses indicate that epiploic WAT exhibits particularly high levels of intercellular communication, with adipocytes actively signalling to myeloid cells [48]. Adipo^*SAA*^ appear central to this network, displaying transcriptional signatures consistent with innate immune modulation rather than classical lipid storage (Fig. 2). This observation challenges the prevailing view that immune activation in adipose tissue is driven predominantly by infiltrating leukocytes and instead implicates adipocytes themselves as orchestrators of local immune responses in specific depots.

The anatomical location of epiploic WAT raises the possibility that microbial-derived signals contribute to shaping this adipocyte state. Experimental evidence indicates that bacterial components, such as lipopolysaccharide, selectively induce SAA1 expression in white adipocytes, consistent with a model in which gut-derived factors act on neighbouring adipose tissue [48]. In this context, Adipo^*SAA*^ may function as sentinels that translate microbial or barrier-related cues into adipocyte-driven immune activation, linking intestinal homeostasis to systemic inflammation. Such depot-specific specialisation has important implications for metabolic and inflammatory disease. Epiploic WAT represents a site where metabolic and immune functions converge, with adipocytes adopting roles that extend beyond energy handling to include regulation of innate immunity. Dysregulation of this adipocyte–immune axis could contribute not only to cardiometabolic disease but also to inflammatory conditions involving the gut, although causal relationships remain to be established.

These findings underscore that adipocyte heterogeneity is not only a property of cell-intrinsic states but is also shaped by anatomical context. Enrichment of immunomodulatory adipocyte populations in specific depots highlights the need to move beyond uniform models of adipose inflammation, towards a spatially informed understanding of how adipose tissue contributes to disease.

## Open science and data accessibility

Progress in human WAT research has historically been constrained by limited access to well-phenotyped tissue samples, heterogeneity in analytical approaches, and the inherent difficulty of reproducing findings across cohorts and laboratories. Many insights have relied on relatively small datasets or specific experimental conditions, making it challenging to assess generalisability or to integrate results across studies. As adipose biology has become increasingly data-rich and multidimensional, these limitations have grown more pronounced.

To address this, recent efforts have focused on creating openly accessible resources that enable systematic exploration of human WAT biology across cohorts, depots and experimental platforms. We developed the Adipose Tissue Knowledge Portal (adiposetissue.org) as a public repository, integrating transcriptomic and phenotypic data from over 60 independent datasets, encompassing >6000 individuals [52]. By harmonising data derived from bulk RNA sequencing, single-cell and single-nucleus transcriptomics, spatial profiling and detailed clinical phenotyping, this resource enables researchers to interrogate adipose tissue biology at multiple levels of resolution. A key strength of such platforms lies in their ability to capture biological diversity. Inter-individual variation, sex differences, depot-specific features and responses to interventions can be explored across studies rather than inferred from isolated cohorts. This is particularly important in WAT research, where subtle differences in tissue composition or metabolic state may have outsized effects on interpretation. Open access to primary data facilitates hypothesis generation, independent validation and cross-study comparison, thereby strengthening mechanistic conclusions. Complementary cross-species analyses are supported through the Mammalian Adipose Tissue Knowledge Portal (MATKP; matkp.org), a publicly available resource developed by an international consortium of academic researchers and hosted by the Broad Institute [Cambridge, MA, USA], which provides access to adipose-related datasets from experimental model systems. Integration of human and animal data allows conserved and species-specific mechanisms to be distinguished more clearly, improving translational relevance while avoiding overgeneralisation from model organisms. Together, these resources aim to promote a bidirectional flow between human discovery and experimental mechanistic work.

Beyond their immediate analytical utility, open data platforms promote collaboration, transparency and independent validation across cohorts. This is particularly important in WAT research, where biological heterogeneity complicates interpretation and generalisation. As the field moves towards increasingly granular descriptions of cellular states and tissue organisation, accessible and well-curated datasets will be essential for reproducible and integrative research.

## Conclusions and future perspectives

Recent advances have established WAT as a metabolically and immunologically active organ, the function of which is shaped by lipid turnover, cellular composition and anatomical context. While these insights have clarified key mechanisms linking adiposity to cardiometabolic disease, they have also exposed important gaps in our understanding of adipose tissue biology. This includes biological diversity related to sex and ethnicity, factors that influence WAT biology in important ways. Adipose tissue distribution differs markedly between men and women, with women generally exhibiting greater subcutaneous storage capacity and relative protection from visceral fat accumulation prior to menopause [53]. Ethnic differences in fat distribution and ectopic lipid deposition are also well documented and influence cardiometabolic risk at comparable BMI levels [54]. How sex, developmental programming and genetic background shape adipocyte turnover, state composition and depot-specific immune interactions remains incompletely understood and represents an important area for future investigation.

A major unresolved question concerns the stability and reversibility of adipocyte states in humans. Longitudinal studies tracking individual adipocyte populations across weight gain, weight loss and pharmacological intervention are required to determine whether maladaptive states can be durably reprogrammed or merely transiently suppressed. Similarly, the molecular mechanisms underlying adipose ‘memory’ remain incompletely defined, including how transcriptional, epigenetic and structural features interact to constrain long-term metabolic outcomes.

Another critical knowledge gap relates to causality. While associations between adipocyte heterogeneity, lipid turnover and inflammation are now reported across multiple studies, direct evidence linking specific adipocyte states to cardiometabolic endpoints in humans is lacking. Addressing this will require integration of deep tissue phenotyping with prospective clinical studies and mechanistic model systems that preserve human-relevant adipocyte biology. Furthermore, the discovery of depot-specific immunomodulatory adipocytes raises new questions regarding adipose–immune communication at barrier sites. Defining how microbial-derived signals, local inflammation and adipocyte state transitions intersect may provide novel insights into the links between metabolic and inflammatory disease. Together, these challenges point towards a shift from weight-centric to tissue-centric therapeutic strategies. Interventions that restore adipocyte function, normalise lipid turnover and rebalance adipocyte states, rather than focusing exclusively on weight loss, may offer more sustained protection against metabolic disease.

Finally, while this review has focused on work from our own group, reflecting the scope of the Camillo Golgi Prize Lecture [55] on which it is based, it is important to emphasise that these findings represent only a small part of a much broader and rapidly advancing field. Major conceptual and technical advances in adipose tissue biology have been driven by the work of many outstanding research groups worldwide. I recognise that important contributors or relevant studies may not be cited as this article is not intended to provide a comprehensive review of the field. Together, this collective body of work has fundamentally reshaped our understanding of WAT as a dynamic, heterogeneous and biologically complex organ, and continued progress will depend on the integration of insights across disciplines, models and research communities.

## Supplementary Information

Below is the link to the electronic supplementary material.

Slideset of figures (PPTX 657 KB)

## Abbreviations

MHO: Metabolically healthy obesity
WAT: White adipose tissue
