# Supplementary material for: Perspectives on human adipose tissue: from cellular mechanisms to clinical complications
Source: Diabetologia. 2026 Apr 24;69(7):1731–40. doi: 10.1007/s00125-026-06735-0 (PMC13236842; doi:10.1007/s00125-026-06735-0)
Supplement: Supplementary file 1 — Slideset of figures (PPTX 657 KB) [file 125_2026_6735_MOESM1_ESM.pptx]

## Slide 1
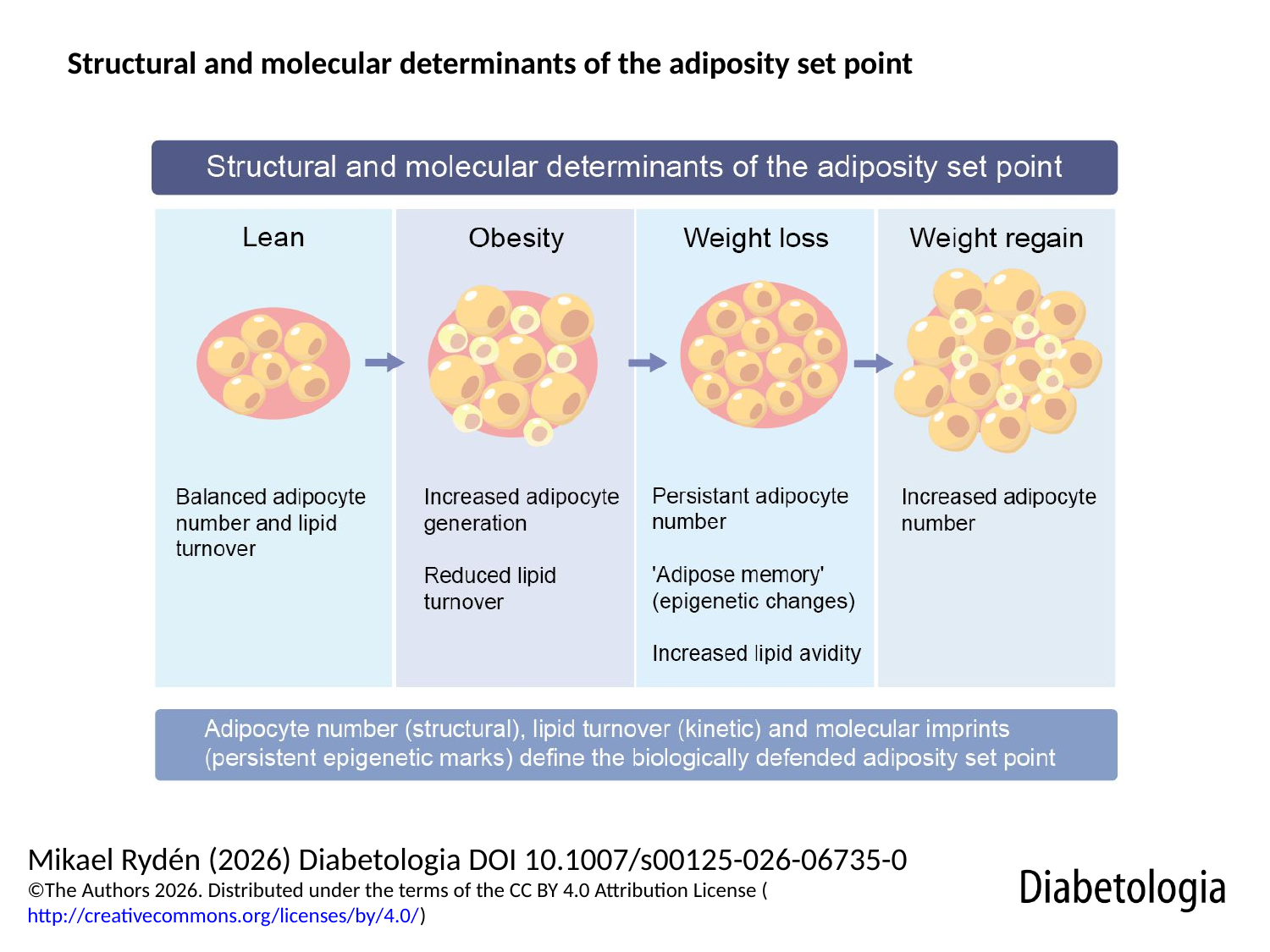

Structural and molecular determinants of the adiposity set point
Mikael Rydén (2026) Diabetologia DOI 10.1007/s00125-026-06735-0
©The Authors 2026. Distributed under the terms of the CC BY 4.0 Attribution License (http://creativecommons.org/licenses/by/4.0/)

## Slide 2
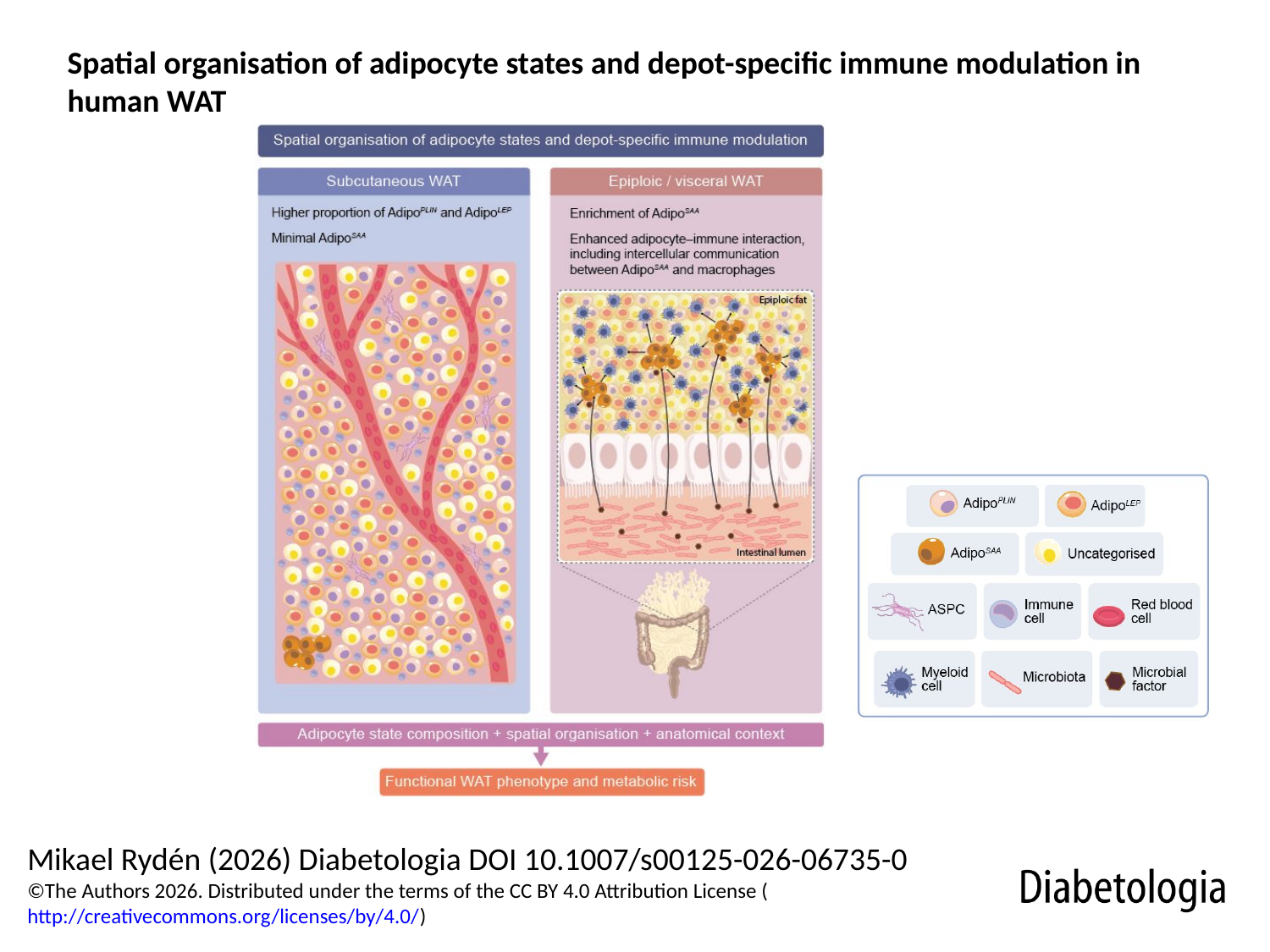

Spatial organisation of adipocyte states and depot-specific immune modulation in human WAT
Mikael Rydén (2026) Diabetologia DOI 10.1007/s00125-026-06735-0
©The Authors 2026. Distributed under the terms of the CC BY 4.0 Attribution License (http://creativecommons.org/licenses/by/4.0/)
